# Early mobilization to prevent ICU-acquired weakness in mechanically ventilated patients: an integrative review

**DOI:** 10.3389/fmed.2026.1808281

**Published:** 2026-06-15

**Authors:** Xinru Song, Ziwei He, Aijian Lei, Yanxin Ma, Yuding Hu, Xiao Li, Cheng Zhang, Pingping Gao, Tianyuan Liu, Kun Zhao, Guoying Wang

**Affiliations:** 1 Department of Intensive Care Medicine, The Second Hospital of Hebei Medical University, Shijiazhuang, Hebei, China; 2 Nursing School of Hebei Medical University, Shijiazhuang, Hebei, China; 3 Department of Emergency, The Second Hospital of Hebei Medical University, Shijiazhuang, Hebei, China

**Keywords:** early mobilization, integrative review, intensive care units, mechanical ventilation, ICU-acquired weakness

## Abstract

**Background:**

Intensive Care Unit-acquired weakness (ICU-AW) is a prevalent and serious complication in critically ill patients, leading to difficulty in weaning from mechanical ventilation, prolonged hospital stays, and impaired functional status after discharge.

**Aim:**

To retrieve, evaluate, and integrate the best evidence for preventing ICU-AW through early mobilization in mechanically ventilated patients, and to provide references for clinical practice.

**Methods:**

Following the “5S” pyramid model, a top-down, systematic retrieval was conducted across domestic and international databases. The types of literature encompass clinical decisions, guidelines, evidence summaries, best practices, expert consensus, group standards, and systematic reviews.

**Results:**

A total of 22 studies were included, comprising 1 evidence summary, 3 guidelines, 3 expert consensus statements, and 15 systematic reviews. 27 pieces of evidence were extracted from these articles, covering 9 areas: establishment of a multidisciplinary rehabilitation team, timing of mobilization, mobilization assessment, mobilization methods, frequency and intensity of exercise, pre-mobilization preparation, mobilization monitoring, post-mobilization observation, and precautions.

**Conclusion:**

This study presents key evidence on early mobilization to prevent ICU-AW in mechanically ventilated patients, highlighting its role in improving patient outcomes. Systematic assessment, timely initiation, individualized mobilization plans, progressive exercise protocols, continuous monitoring, and multidisciplinary collaboration are essential for the effective and safe implementation of mobilization. Early passive and active mobilization can reduce the incidence of ICU-AW, shorten ventilation and ICU stay, and improve functional status at discharge.

**Systematic review registration:**

http://ebn.nursing.fudan.edu.cn/registerResources, identifier ES20245841.

## 1 Introduction

Intensive Care Unit-Acquired Weakness (ICU-AW) is a neuromuscular dysfunction caused by various reasons, primarily characterized by limb weakness and clinical manifestations such as difficulty weaning from mechanical ventilation, diminished reflexes, and muscle atrophy (1). ICU-AW profoundly affects patients by prolonging mechanical ventilation (MV) duration and ICU length of stay (LOS), as well as increasing the economic burden on families. In addition, it can cause persistent physiological, psychological, and cognitive disorders post-discharge, significantly impacting patients’ quality of life (2–4). Mechanical ventilation is a commonly used treatment method in critically ill patients, which is essential for maintaining the patient’s respiratory system function and providing time for disease treatment. However, it should be noted that mechanical ventilation restricts patients’ activities, and mechanical ventilation and inactivity are significant factors in ICU-AW (4). Conversely, ICU-AW prolongs the duration of mechanical ventilation (5). Study shown that 26 to 65% of patients receiving mechanical ventilation develop ICU-AW within 5 to 7 days (6). Moreover, the incidence of ICU-AW in patients receiving prolonged MV for at least 10 days reaches 67% (6).

The patients in the ICU are suffering from severe conditions that are constantly changing, with extended treatment cycles. Due to long-term immobility and bed rest, muscle mass decreases by more than 10% after the first week of ICU admission, and the decrease in muscle mass is a significant factor in ICU-AW (7). The pathophysiological mechanisms underlying ICU-AW remain incompletely understood, and effective treatment options are currently limited globally (4). Early mobilization has been demonstrated to reduce the duration of MV and LOS (8). Moreover, it is beneficial to enhance muscle strength and immune function. It can also reduce the incidence of ICU-AW, delirium, and deep vein thrombosis of the lower extremities (9, 10). However, a recent multicenter randomized controlled trial reported that its results were not entirely consistent with these conclusions (11). Systematic reviews and randomized controlled trials on early mobilization have been published both domestically and internationally; the evidence remains scattered and does not provide comprehensive, systematic guidance. Moreover, there is a lack of guidelines, expert consensus, and evidence summary studies. Consequently, there is an urgent need to develop comprehensive, scientific, and standardized management programs. This study employed an evidence-based approach, evaluating and integrating evidence on early mobilization in MV patients to synthesize the evidence. It will provide evidence-based guidance for medical staff to develop a scientific, standardized early mobilization program.

## 2 Methods

This evidence summary adheres to the standardized reporting framework proposed by the Fudan University Center for Evidence-based Nursing (12). This standard covers the entire process, from problem identification, evidence retrieval, and literature screening to critical appraisal, evidence synthesis, grading, and the formulation of practice recommendations.

### 2.1 Problem identification

The first step in the evidence summary report of the Fudan University Evidence-Based Nursing Center is “Problem identification” (13). This study formulated the following evidence-based problems following the PIPOST model: the first P (Target Population for Evidence Application) is patients receiving MV during their ICU stay who are≥18 years of age. I (intervention): early mobilization; the second P (professionals applying the evidence): Medical staff in the intensive care unit, physiotherapists. O (outcome): primary outcome: incidence of ICU-AW; secondary outcome: duration of MV, ICU length of stay; S (place of application of evidence): intensive care unit; T (study type): clinical decisions, guidelines, evidence summaries, best practices, expert consensus, group standards, and systematic reviews.

### 2.2 Search strategy

According to the “5S” evidence pyramid model (14), a top-down retrieval of the following databases was performed: UpToDate, BMJ Best Practice, Joanna Briggs Institute (JBI), Guidelines International Network (GIN), National Institute for Health and Care Excellence (NICE), Scottish Intercollegiate Guidelines Network (SIGN), Yi Maitong Guidelines Network, MedSci, Ding Xiang Yuan, Registered Nurses Association of Ontario (RANO), Society of Critical Care Medicine (SCCM), Chinese Nursing Association, the Cochrane Library, PubMed, Web of Science, Embase, CNKI, Wan Fang database, VIP database, SinoMed. This study searched the literature published from January 2014 to December 2024.

**Figure 1:**
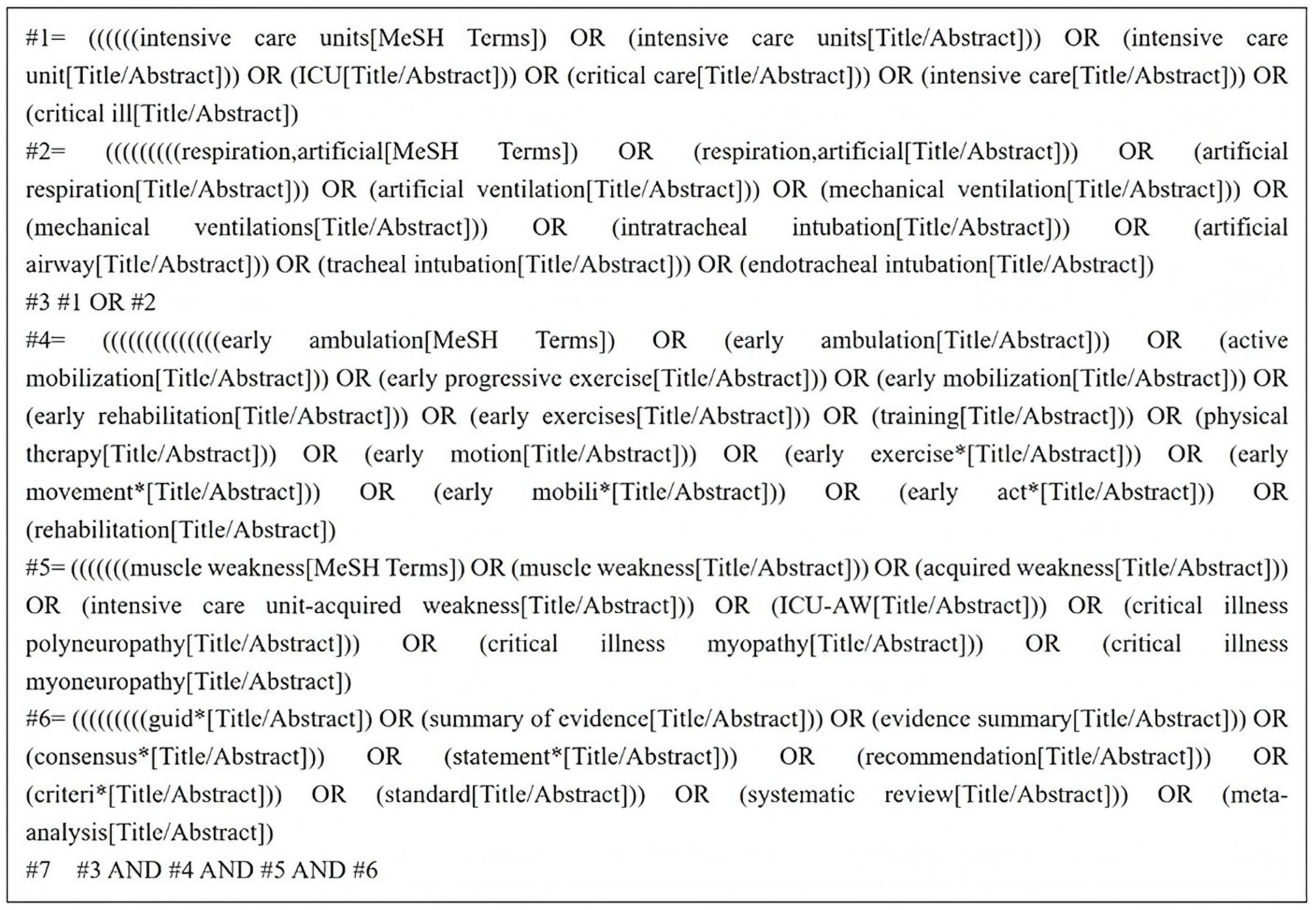
PubMed search strategy.

Retrieval of evidence was performed using subject headings and free words. The search terms were listed as follows: “intensive care units/intensive care unit/ICU/critical care/intensive care/critical ill” “artificial respiration/artificial ventilation/mechanical ventilation/mechanical ventilations/intratracheal intubation/artificial airway/tracheal intubation/endotracheal intubation” “early ambulation/active mobilization/early progressive exercise/early mobilization/early rehabilitation/early exercises/training/physical therapy/early motion/early exercise*/early movement*/ early mobile*/early act*/rehabilitation” “muscle weakness/acquired weakness/intensive care unit-acquired weakness/ICU-AW/critical illness polyneuropathy/critical illness myopathy/critical illness myoneuropathy”. The PubMed search strategy is shown in Figure 1.

### 2.3 Inclusion and exclusion criteria

Inclusion criteria: (1) The study subjects are patients receiving MV in the ICU, ≥18 years of age. (2) Studies related to early mobilization in MV patients; (3) Study types included clinical decisions, clinical guidelines, evidence summaries, best practices, expert consensus, group standards, and systematic reviews; and (4) The languages of research were Chinese and English literature.

Exclusion criteria: (1) The literature was a draft, abstract, or conference paper; (2) Unable to acquire full-text literature; (3) Republished or updated papers; (4) Studies that failed to meet the quality evaluation criteria; and (5) Interpretation of guidelines; translated versions and drafts of guidelines.

### 2.4 Literature screening

All retrieved literature was imported into EndNote 21, and duplicates were removed. Two authors who had received training in the evidence-based nursing system independently conducted the literature screening. The title, abstract, and keywords of the literature were reviewed during the initial screening, and then the full text was read in the rescreening. A quality evaluation of the final piece of literature included was conducted.

### 2.5 Quality evaluation of the literature

The Appraisal of Guidelines for Research and Evaluation System II (AGREE II) (15) was used to evaluate the Guidelines. The evaluation criteria comprise 6 dimensions and 23 items, and each item is rated on a scale of 1–7 (1 indicates complete inconsistency and 7 indicates complete consistency). Standardized score for each field = [(actual score for each field − possible lowest score)/(possible highest score − possible lowest score)] × 100%. Higher standardized percentages of scores for each dimension indicate a higher quality of the guideline. A standardized score was calculated for each dimension to classify the guidelines into three levels. 6 dimensions scoring ≥60% were designated as level A, strongly recommended; a count of dimensions scoring ≥30 and <60% as level B, recommended; a count of dimensions scoring <30% as level C, not recommended.

The Expert Consensus Evaluation Criteria of JBI Evidence-Based Health Care Centers (2016 edition) were used to evaluate expert consensuses. This tool consists of six fields, each with four choices: “Yes”, “No”, “Unclear”, and “Not Applicable” (16).

The systematic review tool proposed by the Australian JBI Center for Evidence-Based Health Care (17) was used to evaluate the quality of the systematic reviews. The tool consists of 11 evaluation items, each containing four choices: “Yes”, “No”, “Unclear”, and “Not Applicable”.

The quality evaluation of the evidence summary was conducted by tracing the primary articles and selecting the appropriate evaluation criteria.

Two researchers who have received evidence-based nursing system training independently evaluated the included literature. If the results were inconsistent, a third evidence-based expert was invited to discuss whether to include the literature in the analysis.

### 2.6 Evidence extraction and summary

Evidence extraction was conducted independently by two researchers, who had received systematic training in evidence-based nursing, including the source of literature, study type, publication year, topic of study, and evidence content. After extraction, two researchers conducted cross-checks and sorted and summarized the data by theme. Evidence synthesis should adhere to the following principles: (1) if the content was complementary, they were merged based on the logical relationship of the language; (2) when the content was consistent, simple and straightforward language was used; (3) when the content was independent, the original content was retained; and (4) when there are differences in the contents of the evidence, the principle of giving priority to evidence-based evidence, high-quality evidence and the latest published evidence would be followed for selection.

This study adopts the JBI evidence pre-classification and evidence recommendation level system (2014 version) to classify the included evidence. Evidence was categorized into levels 1 to 5 according to study type, with level 1 representing the highest quality and level 5 the lowest (18). An expert consensus statement reflects the best clinical practice when evidence is insufficient. When discrepancies arose in the evaluation opinions, the third expert was invited to the discussion and eventually reached a consistent conclusion. The feasibility, suitability, clinical significance, and effectiveness of the evidence were discussed. The suggestion, in combination with the JBI evidence recommendation levels of the 2014 version, is divided into A-level recommendations (strong recommendations) and B-level recommendations (weak recommendations).

### 2.7 Study registration

http://ebn.nursing.fudan.edu.cn/registerResources, identifier ES20245841.

## 3 Results

### 3.1 Search results

**Figure 2:**
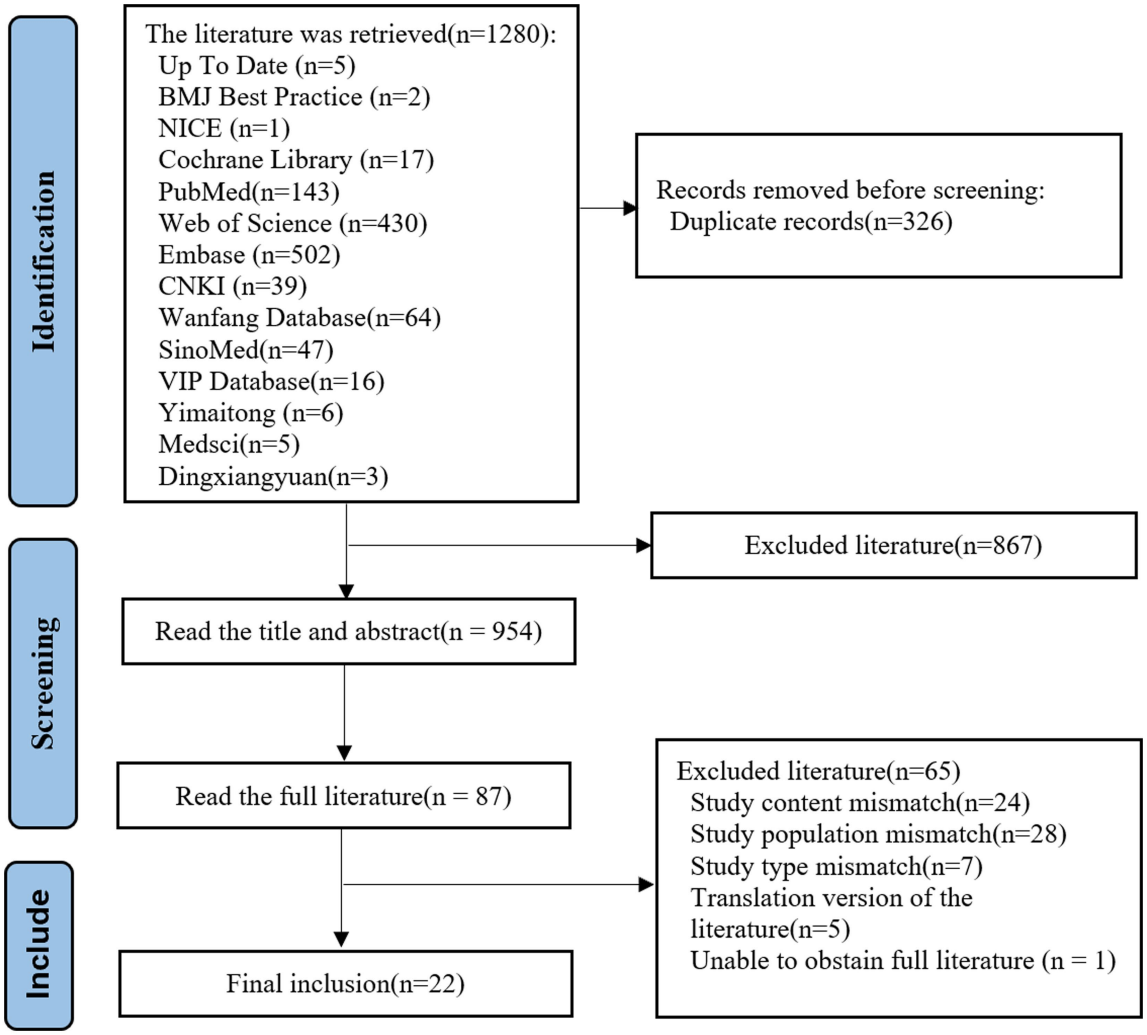
Literature screening flowchart.

A total of 1,280 articles were identified, and 22 studies were included after removing duplicates, reading titles and abstracts, and reading the full text. The screening process is shown in Figure 2. The included studies comprised 3 clinical guidelines (1, 19, 20), 3 expert consensus statements (21–23), 15 systematic reviews (8–10, 24–35), and 1 evidence summary (36). The general characteristics of the included studies are shown in Table 1.

**Table 1:** Characteristics of included studies (*n* = 22).

Included literature	Year of publication (year)	Source	Type of literature	The literature theme
Aquim et al. (19)	2019	PubMed	Guideline	Brazilian guidelines for early mobilization in intensive care unit
Green et al. (20)	2016	PubMed	Guideline	Mobilization of intensive care patients: a multidisciplinary practical guide for clinicians
Fan et al. (1)	2014	PubMed	Guideline	An Official American Thoracic Society Clinical Practice Guideline: The Diagnosis of Intensive Care Unit-acquired Weakness in Adults
Wu et al. (22)	2018	Wanfang database	Expert Consensus	Expert consensus on rehabilitation treatment techniques for respiratory critical care in China
Wang et al. (23)	2017	Wanfang database	Expert Consensus	Expert consensus on critical care rehabilitation in Zhejiang Province
Raurell-Torredà et al. (21)	2021	PubMed	Expert Consensus	Early mobilisation algorithm for the critical patient. Expert recommendations
Sun et al. (36)	2024	Wanfang database	Summary of evidence	Summary of the best evidence for early exercise rehabilitation in patients with mechanically ventilated ICU-acquired weakness
Wang et al. (10)	2021	CNKI	Systematic review	Application effect of early in-bed cycling exercise in mechanically ventilated ICU patients: a Meta-analysis
Hu et al. (24)	2020	CNKI	Systematic review	Effect of early mobilization on ICU-acquired weakness in patients with mechanical ventilation: cumulative Meta-analysis and trial sequential analysis
Zhao et al. (25)	2017	CNKI	Systematic review	The effects of early progressive exercise on ICU-acquired weakness: a meta-analysis
Yang et al. (26)	2017	CNKI	Systematic review	The effect of early active mobilization for patients with mechanical ventilation in intensive care unit: a meta analysis
Wang et al. (9)	2023	Wanfang database	Systematic review	Effect of early systemic rehabilitation on muscle strength and prognosis of patients undergoing mechanical ventilation in intensive care unit: a Meta-analysis
Cheng et al. (27)	2021	Wanfang database	Systematic review	Meta-analysis of the effects of neuromuscular electrical stimulation of lower limbs on patients with mechanical ventilation in the intensive care unit
Ding et al. (28)	2019	Wanfang database	Systematic review	Safety criteria for early goal-oriented rehabilitation exercise in patients undergoing mechanical ventilation in the intensive care unit: a systematic review
Liang et al. (29)	2019	Wanfang database	Systematic review	Meta-analysis of the intervention effects of early ambulation on acquired weakness in intensive care unit patients with mechanical ventilation
Ruo Yu et al. (30)	2024	PubMed	Systematic review	Optimal timing for early mobilization initiatives in intensive care unit patients: A systematic review and network meta-analysis
Li et al. (31)	2024	PubMed	Systematic review	The effect of electrical stimulation in critical patients: a meta-analysis of randomized controlled trials
Wang et al. (8)	2023	PubMed	Systematic review	The effects of early mobilization in mechanically ventilated adult ICU patients: systematic review and meta-analysis
Liu et al. (32)	2020	PubMed	Systematic review	Intervention effect of neuromuscular electrical stimulation on ICU-acquired weakness: a meta-analysis
Clarissa et al. (33)	2019	PubMed	Systematic review	Early mobilisation in mechanically ventilated patients: a systematic integrative review of definitions and activities
Ding et al. (34)	2019	PubMed	Systematic review	What is the optimum time for initiation of early mobilization in mechanically ventilated patients? A network meta-analysis
Doiron et al. (35)	2018	PubMed	Systematic review	Early intervention (mobilization or active exercise) for critically ill adults in the intensive care unit

### 3.2 Literature quality evaluation results

#### 3.2.1 Quality evaluation results of guidelines

This study included three guidelines (1, 19, 20) and evaluated their quality using AGREE II. Three guidelines were rated A-level due to standardized scores of 60% or higher in each field, indicating overall high quality. The quality evaluation results are shown in Table 2.

**Table 2:** Quality evaluation results of the included guidelines (*n* = 3).

Inclusion guidelines	Standardized scores in various domains (%)						≥60%	≥30%	Quality grade
	Scope and purpose	Stakeholder involvement	Rigor of development	Clarity	Applicability	Independence			
Aquim et al. (19)	100.00	83.33	89.58	100.00	58.33	66.67	6	6	A
Green et al. (20)	97.22	83.33	61.46	100.00	83.33	70.83	6	6	A
Fan et al. (1)	94.44	94.44	89.58	86.11	60.42	66.67	6	6	A

#### 3.2.2 Quality evaluation results of expert consensus

Three expert consensus were encompassed and evaluated for quality. The three expert consensus statements (21–23) were rated as high quality and included. The evaluation results are presented in Table 3.

**Table 3:** Quality evaluation of included expert consensus.

Items	Wu et al. (22)	Wang et al. (23)	Raurell-Torredà et al. (21)
1. Is the origin of the viewpoint clearly labeled?	Yes	Yes	Yes
2. Are the concepts derived from authoritative experts in the field?	Yes	Yes	Yes
3. Are the viewpoints proposed centered on the population’s interests relevant to the study?	Yes	Yes	Yes
4. Is the stated conclusion the process according to the analysis? Is the expression of viewpoints logical?	Yes	Yes	Yes
5. Is there a reference to other existing literature?	Yes	Yes	Yes
6. Are there any inconsistencies between the viewpoints proposed and the existing literature?	Unclear	No	Unclear

#### 3.2.3 Quality evaluation results of systematic reviews

Fifteen systematic reviews were included and evaluated for quality. The evaluation results are presented in Table 4.

**Table 4:** Quality evaluation of included systematic reviews.

Included literature	P1	P2	P3	P4	P5	P6	P7	P8	P9	P10	P11
Wang et al. (10)	Y	Y	Y	Y	Y	Y	Y	Y	U	Y	Y
Hu et al. (24)	Y	Y	Y	Y	Y	Y	Y	Y	Y	Y	Y
Zhao et al. (25)	Y	Y	Y	Y	Y	Y	Y	Y	U	Y	Y
Yang et al. (26)	Y	Y	Y	Y	Y	Y	Y	Y	Y	Y	Y
Wang et al. (9)	Y	Y	Y	Y	Y	Y	Y	Y	Y	Y	Y
Cheng et al. (27)	Y	Y	Y	Y	Y	Y	Y	Y	Y	Y	Y
Ding et al. (28)	Y	Y	Y	Y	Y	Y	Y	Y	Y	Y	Y
Liang et al. (29)	Y	Y	Y	Y	Y	Y	Y	Y	N	Y	Y
Ruo Yu et al. (30)	Y	Y	Y	Y	Y	Y	Y	Y	Y	Y	Y
Li et al. (31)	Y	Y	Y	Y	Y	Y	Y	Y	Y	Y	Y
Wang et al. (8)	Y	Y	Y	Y	Y	Y	Y	Y	U	Y	Y
Liu et al. (32)	Y	Y	Y	Y	Y	Y	Y	Y	Y	Y	Y
Clarissa et al. (33)	Y	Y	Y	Y	Y	Y	Y	Y	N	Y	Y
Ding et al. (34)	Y	Y	Y	Y	Y	Y	Y	Y	Y	Y	Y
Doiron et al. (35)	Y	Y	Y	Y	Y	Y	Y	Y	U	Y	Y

Y: Yes, N: No, U: Unclear. P1: Are the study questions raised clearly and definitively? P2: Are the inclusion criteria of the articles appropriate? P3: Is the retrieval strategy appropriate? P4: Is the literature source appropriate? P5: Are the adopted literature quality evaluation criteria appropriate? P6: Is the quality of the literature evaluated independently by two or more researchers? P7: Are some measures applied to minimize errors when extracting data? P8: Are the methods of combined studies appropriate? P9: Is the possibility of publication bias assessed? P10: Whether to make recommendations for policy and/or practice regarding reporting data? P11: Are appropriate suggestions put forward for the specific directions of further research in the future?

### 3.3 Summary of evidence

After rigorous literature retrieval, quality evaluation, and evidence integration, the relevant evidence to prevent ICU-AW during mechanical ventilation was identified. The study summarized 27 recommendations, including the establishment of a multidisciplinary team, timing of mobilization, mobilization assessment criteria, mobilization methods, frequency and intensity of exercise, pre-mobilization preparation, exercise monitoring, post-exercise observation, and precautions, which were divided into nine areas, as shown in Table 5.

**Table 5:** Best evidence summary of early mobilization interventions for preventing ICU acquired weakness in mechanically ventilated patients.

Category	Content of evidence	Level	Recommendation level
Establish a multidisciplinary team	1. Establish a multidisciplinary team (MDT), composed of experienced critical care medicine doctors and nurses, rehabilitation doctors, respiratory therapists, physical therapists, and nutritionists. Team members must receive training related to early mobilization (21–23, 29, 33).	1	A
Timing of mobilization	2. Once the patient is admitted to the intensive care unit, the multidisciplinary team can assess the patient’s condition, and an early mobilization program can be formulated. Implementation mobilization within 24~72 h of mechanical ventilation may be optimal (30, 34).	1	A
Mobilization assessment	3. The MDT is accountable for identifying the indications and contraindications for early mobilization (19, 20, 28).	1	A
	4. It is recommended that the RASS score of patients be within (−1 to 1) points during early mobilization intervention (36).	1	A
	5. Indications (19, 22, 28, 36). (1) Hemodynamic stabilization: systolic blood pressure (SBP) > 90 mmHg and<170 mmHg. (2) 65 mmHg ≤ mean arterial pressure (MAP) ≤ 110 mmHg. (3) Heart rate (HR): HR > 40 bpm and<120 bpm. (4) Respiratory stabilization:peripheral oxygen saturation (SpO_2_) ≥ 90%, fraction of inspired oxygen (FiO_2_) ≤ 60% and respiratory rate (RR) ≤ 25irpm, positive end-expiratory pressure (PEEP≤10 cmH_2_O).	1	A
	6. Contraindication (19, 23, 36): intracranial hypertension, hemodynamic instability, unstable fractures, recent acute myocardial infarction, open abdominal wounds, deep vein thrombosis.	1	A
	7. Patients were jointly evaluated by doctors, physical therapists, and nurses, and the sedation Score (RASS) was used to assess the patient’s level of consciousness (21). Muscle strength is assessed using the MRC scale, and uncooperative patients are tested with electrophysiologic tests; ultrasound may also be used to monitor muscle changes (20, 21).	1	A
	8. A comprehensive evaluation should be conducted to minimize the patient’s potential risks. If any are concerned that certain factors may affect the patient’s early mobilization, it will be discussed with the physical therapist and physician (20).	1	A
Mobilization methods	9. Exercise should be implemented gradually based on the patient’s condition. It is recommended that this be performed cooperatively by doctors, rehabilitation therapists, and nurses (10, 21, 22, 24, 25, 31–33). (1) Passive activities encompass daily awakening, turning over, passive flexion and extension of limb joints, bed cycling exercises, and neuromuscular electrical stimulation, which are suitable for patients in a sedated state or with consciousness disorders. (2) Assisted activities include joint-assisted mobilization, assisted bedside sitting, stepping apparatus-assisted ambulation. (3) Active activities include abdominal breathing training, active joint movement, bedside sitting training, independent walking, and resistance training, which are suitable for conscious patients.	1	A
	10. Formulate a graded activity plan and select passive, assisted, and active exercise methods according to the patient’s condition (20, 23, 31, 32). (1) For patients who are unconscious or unable to cooperate actively, a first-level movement mode is adopted, including neuromuscular electrical stimulation therapy and turning over every 2 h. (2) Muscle strength < grade 3: Use a two-level exercise method. Except for turning over, the patient’s joint range of motion should be maintained to prevent muscle atrophy. Place the patient in an appropriate limb position. Patients should maintain a sitting posture for at least 20 minutes, three times a day. (3) Upper arm muscle strength ≥ grade 3: Adopt a grade 3 exercise mode, and the patient sits on the edge of the bed. (4) Lower limbs≥ grade 3: A grade 4 exercise method is adopted. The patient stands or sits in a wheelchair and maintains a sitting position for at least 20 minutes every day. (5) The five-level movement mode should gradually reach the stage where they can get out of bed and walk.	5	A
Frequency and intensity of exercise	11. Exercise intensity: The intensity of exercise should take into account clinical efficacy, individual tolerance, age, and previous conditions (19, 20).	1	A
	12. The specific intensities are as follows (10, 19, 31, 32). (1) Bed bicycle activity: up to 1 h per day, twice a day. (2) Passive joint movement: 10 to 20 mobilizations per joint, up to two times/day. (3) Neuromuscular electrical stimulation (NMES): 30 to 60 minutes a day, 1 to 2 times a day, starting at low frequencies (30- 50 Hz). (4) Active exercise: flexion, inward and outward rotation of the upper extremities; weight shifting on both sides of the body; forward and backward body movement; and trunk rotation in a seated position, two 30-minute sessions per day. (5) If conditions permit, a standing bed can be used to assist in standing upright for 30 minutes twice a day.	1	A
Pre-mobilization preparation	13. The early rehabilitation team conducts case discussions, has a clear division of labor within the team, communicates and coordinates, and determines a leader (20).	1	A
	14. Inform patients and their families of the benefits and risks of early mobilization, sign the informed consent form, and answer the doubts of conscious patients (20).	1	A
	15. Comprehensive assessment of the patient according to safety criteria before each start of early activity (28).	1	B
	16. Number of personnel: Three to five staff members were required, with one member specifically for airway management (20, 33).	1	A
	17. Preparation of material: remove unnecessary equipment and check necessary items for the activity, such as suction devices, oxygen, infusion stands, and bicycles (21).	5	B
	18. Ensure appropriate plumbing infrastructure is in place. It is recommended that the disconnection of redundant pipelines be considered (21).	5	B
	19. Clear the airway: Suctioning should be performed as required, and a vibrating sputum expectorator may be considered if deemed necessary (20, 22).	1	A
	20. Before the activity, a backup plan should be made. If the patient is unable to complete the initial plan, the backup plan may be activated (20).	1	A
	21. Allocate 30–60 minutes(including preparation time)for mobilization of mechanically ventilated patients (20).	1	A
Exercise monitoring	22. During the implementation of the exercise, nurses closely monitored patients’ vital signs and parameters of the ventilator (19–22).	1	A
	23. Timing of cessation of exercise (21–23, 28). (1) SBP > 180 mmHg OR DBP > 110 mmHg, MAP<65 mmHg OR >120 mmHg, compared with before the activity, the change in SBP was more than 20%. (2) HR<40 bpm OR >130 bpm, decrease > 20% based on resting heart rate. (3) Respiratory rate > 30/min OR have difficulty breathing, SpO_2_<88%, FiO_2_ ≥ 60%, PEEP>10cmH_2_O. (4) Severe cardiac arrhythmia, acute myocardial infarction, sepsis, gastrointestinal hemorrhage, chest pain.	5	B
Post-mobilization observation	24. Accurately record the changes in equipment parameters, etc., and actively prevent adverse events such as unplanned extubation (10, 21).	5	A
Precautions	25. Muscle mass can be measured by ultrasound. The functional status and muscle strength of the patients were measured using the total score of MRC, the Chelsea Intensive Care Physical Therapy Scale (CPAx), the Intensive Care Physical Fitness Test (PFIT), and the ICU Activity Scale (IMS) (1, 21, 23).	1	A
	26. Ensuring that the surrounding environment is safe and clean, that the equipment and devices are in normal working condition, and that sufficient trained personnel are available for deployment in case of emergencies (28).	1	A
	27. The multidisciplinary team should establish emergency plans for early mobilization, such as unplanned extubation, bed falls, and falls. In case of adverse events, handle them promptly and report them (19).		

## 4 Discussion

### 4.1 Evidence formation

The PIPOST principle was adopted to formulate evidence-based questions. Adhering to the “5S” pyramid model (14), this study searched for evidence in domestic and international databases and on relevant professional society websites. Rigorous literature screening and quality evaluation were conducted in accordance with scientific, rigorous, and transparent research principles. Appropriate and convincing evaluation tools were applied to different types of literature to ensure the reliability of the quality evaluation. Two authors, who had received systematic training in evidence-based nursing, independently conducted literature retrieval, screening, quality evaluation, and synthesis of evidence. In the event of any discrepancies, a third expert was consulted to decide on inclusion. Additionally, this project established a multidisciplinary team comprising doctors, nurses, and rehabilitation therapists to assess feasibility, suitability, clinical significance, and effectiveness. After extracting the evidence, the research team used the JBI evidence pre-grading system to grade it (18). The recommendation level was determined based on the FAME structure of JBI evidence (18). The evidence formation process is both scientific and rigorous, providing reliable guidance for clinical practice.

### 4.2 Multidisciplinary collaboration

Multidisciplinary treatment (MDT) is a diagnosis-and-treatment model centered on patients and characterized by close collaboration among multiple team members. By formulating scientific, standardized, and precise comprehensive treatment plans, it can compensate for the deficiencies existing in single-specialty treatments and break down professional barriers. Good teamwork is a scientific approach that can facilitate the early mobilization of ICU patients and guide the team’s work (37). The MDT model is now widely applied in the ICU for critically ill patients, and the safety of early mobilization is closely tied to the cooperation among the team members. Green et al. (20) recommended that early mobilization be conducted by an interdisciplinary team, which includes medical, nurses, respiratory therapists, psychotherapists, nutritionists, and physiotherapy staff. The multidisciplinary team collaborates to provide comprehensive care, including systematic assessment and timely initiation of therapy, which contributes to improved long-term quality of life in patients. During early mobilization, nurses can play a leading role. As the 24-h bedside caregivers, they can observe changes in patients’ conditions. They should not passively wait for doctors’ orders but should actively assess and propose early mobilization to doctors.

To ensure the consistency, safety, and efficacy of the early mobilization, medical staff should receive standardized training. This training included the criteria for initiating and suspending early mobilization, role assignment, and emergency response procedures. Wang et al. (38) employed a self-designed questionnaire to investigate the knowledge, attitudes, and practices regarding ICU-AW among 249 ICU nurses. The level of knowledge, attitudes, and practices among nursing staff regarding ICU-AW is low, and most nurses showed a positive attitude and were willing to accept relevant training. Studies have shown that training effectively enhances ICU nurses’ knowledge, beliefs, and behaviors regarding ICU-AW (39). In the implementation stage of evidence conversion in early mobilization, it is crucial to ensure that medical staff fully grasp relevant knowledge, such as risk factors and assessment measures, and understand the importance of early mobilization of ICU patients, thereby promoting the effective implementation and application of evidence. Moreover, the hospital management should provide the personnel and material conditions to implement EM (40).

### 4.3 Comprehensive assessment

The implementation of early mobilization in ICU is not simply about patients “moving”; it is a systematic safety management process that runs throughout the entire ICU stay. The patients in the ICU are suffering from severe conditions that are constantly changing. Comprehensive assessment and preparation in the early stage are crucial for ensuring the safety of MV patients and for determining whether patients on mechanical ventilation can successfully perform early mobilization and benefit from it. The multidisciplinary team should comprehensively assess patients upon admission or when their condition changes, retaining the necessary central venous catheter and artificial airway to minimize catheter-related risks (21). Early assessment primarily involves the cardiovascular, respiratory, nervous, and orthopedic systems (28, 40). The advantage of timely assessment is that the intensity, duration, and frequency of mobilization can be adjusted based on the patient’s condition and tolerance level. High-risk factors identified in the assessment should be addressed as early as possible in the preparation phase to minimize the incidence of adverse events.

The most widely used muscle strength assessment tool is the Medical Research Council (MRC) score, which evaluates the strength of six muscle groups bilaterally to obtain a total score. If the total score is less than 48, it is diagnosed as ICU-AW (41). However, because MRC is only applicable to patients who are awake and able to cooperate, electrophysiological examination or ultrasound can be used for unconscious patients. In addition, the Chelsea Critical Care Physical Assessment Tool (CPAx) not only assesses body mobility and physical function using other scales but also includes grip strength, respiratory function, and cough ability (42). It can dynamically assess and monitor the physical function status of ICU-AW patients.

### 4.4 Personalized mobilization

In clinical practice, it is crucial to determine the optimal start time for early mobilization and to implement effective, individualized mobilization methods based on the patient’s tolerance and the multidisciplinary team’s diagnosis and treatment plans. Network meta-analysis (30) shows that starting early mobilization 24 ~ 72 h after mechanical ventilation may be an appropriate choice to reduce the incidence of acquired weakness in the ICU and shorten the duration of MV. This result aligns with the recommendations of the Guideline on positioning and early mobilization in the critically ill by an expert panel (40).

Functional mobility typically follows a stepwise manner, from in-bed exercises to sitting at the edge of the bed, followed by transfers and standing, marching in place and ultimately ambulation (43). Due to the variability of MV patients in terms of age, disease type, and health status, the medical team still needs to comprehensively assess the patient’s degree of tolerance to determine the start time of early mobilization, to select the appropriate mode of mobilization, the frequency, and the length of mobilization, and to develop an individualized mobilization program. Physiotherapists are responsible for formulating specific activity prescriptions and technique guidance. Passive activities include daily awakening, turning over, passive flexion and extension of limb joints, bed cycling exercises, and neuromuscular electrical stimulation, and active activities include abdominal breathing training, bridge exercises, joint active movement, bedside sitting training, independent walking, and resistance training (10, 25, 28, 31, 35). Currently, the early rehabilitation of MV patients has evolved beyond a single intervention, and a more diverse range of strategies has been adopted to develop individualized treatment plans and implement early functional training and professional physiotherapy, thereby promoting their rehabilitation (44, 45). Research (46) shows that a combined, multidisciplinary, five-step progressive mobility program based on patients’ muscle strength and specific conditions strengthens muscles, improves MRC scores, reduces ICU-AW and delirium rates, and shortens LOS. Future studies can combine patients’ specific conditions to explore more appropriate forms of early mobilization. Additionally, Schaller et al. (40) recommend integrating early mobilization into the ABCDEF bundle, which includes the management of pain, anxiety, agitation, and delirium, as well as spontaneous breathing trials in mechanically ventilated patients.

### 4.5 Monitoring during mobilization

Due to prolonged bed rest, activity limitations, and a decline in muscle strength, the normal activity function of ICU mechanically ventilated patients is affected, and adverse events such as decreased oxygen saturation, extubation, and falls may occur during mobilization. Studies have shown that the incidence of adverse events during early mobilization is 2.6% (47). All-round monitoring during early mobilization can help medical staff assess patients’ tolerance and serve as a reference for dynamically adjusting the mobilization plan. At the same time, ensure that first-aid equipment, commonly used resuscitation drugs, and negative-pressure suction devices are ready for use to protect patient safety. Moreover, variability remains in the specific quantitative parameters reported in this study; therefore, multidisciplinary teams should individualize decisions based on patients’ diagnosis, muscle strength, and tolerance. It is essential to formulate precautions and appropriate contingency plans to prevent adverse events and to handle them promptly.

## 5 Limitations

This study synthesized evidence on early mobilization to prevent ICU-AW in critically ill patients. However, most of the included evidence was derived from studies conducted in different countries and healthcare systems, which may limit its direct applicability to specific clinical settings. The evidence should be adapted to the local clinical context, available resources, and patients’ preferences. Healthcare professionals are encouraged to use the evidence identified in this review as a flexible framework for context-specific implementation. In addition, this study only included English and Chinese literature, and relevant studies in other languages may have been missed.

## 6 Conclusion

This integrative review synthesized current evidence on early mobilization to prevent ICU-AW in mechanically ventilated patients and developed a structured, clinically applicable framework for implementation in intensive care settings. The findings indicate that early mobilization is not a single intervention, but a structured, multidisciplinary process involving multidisciplinary collaboration, appropriate timing, comprehensive assessment, progressive mobilization methods, continuous monitoring, and precautions. By integrating these components, this review provides a practical approach to support the translation into clinical practice and optimize rehabilitation strategies for critically ill patients. This approach may reduce ICU-AW, shorten the duration of mechanical ventilation and ICU length of stay, and improve functional recovery at discharge. However, further high-quality, multicenter studies are needed to clarify the optimal intensity, frequency, duration, and progression of early mobilization, as well as to explore tailored strategies for different patient subgroups.
